# How Do Men Who Post Publicly on Social Media Author Themselves and Their Experiences of Crohn’s Disease? A Dialogical Analysis of Three Cases

**DOI:** 10.1177/10497323241287453

**Published:** 2024-10-25

**Authors:** Lucy Prodgers, Brendan Gough, Anna Madill

**Affiliations:** 1 Leeds Institute of Health Sciences, School of Medicine, 4468 University of Leeds, Leeds, UK; 2 School of Humanities and Social Sciences, Leeds Beckett University, Leeds, UK; 3 School of Psychology, 4468 University of Leeds, Leeds, UK

**Keywords:** chronic illness, Crohn’s disease, social media, dialogical analysis, men

## Abstract

Despite distinct sex- and gender-related differences in the presentation and manifestation of Crohn’s disease (CD), little research to date has considered men’s particular experiences. Whilst hegemonic masculine ideals have been reported to negatively impact men’s mental and physical health, increasingly research has emphasized that men engage in a diverse range of practices, including those beneficial to health. One such practice is posting about their illness experiences on social media. The interactive nature of posting online means that a dialogical approach, based on a relational epistemology, is particularly useful. This study therefore asked: “How do men who post publicly on social media author themselves and their experiences of CD?” Three participants were recruited, all of whom had a diagnosis of CD, wrote a blog, and posted on other social networking sites (SNSs) about CD. Two resided in Canada and one in the United Kingdom. All were white. For each participant, 2 years of multimodal social media data was downloaded. After screening, in-depth analysis was conducted using a dialogical approach focusing on three key dialogical concepts: genre, chronotope, and forms of authorship. The key findings emphasized the participants’ different responses to the lack of predictability caused by CD and the different ways they used social media to gain a greater sense of control over their illness stories and identities, providing important insights into the interaction between masculine identities and illness. Finally, the potential deployment of such methods in future research and within therapeutic contexts was considered.

## Introduction

Crohn’s disease (CD), a form of inflammatory bowel disease (IBD), is an incurable, immune-related condition which causes inflammation and ulceration in the lining of the gut, leading to symptoms such as severe abdominal pain, diarrhea, and fatigue. It is currently estimated that in the United Kingdom, around 276 people in every 100,000 have the disease (Pasvol et al., 2020), with individuals typically diagnosed prior to the age of 30 (Crohn’s and Colitis UK, 2021).

There is a very slight female predominance of CD in Western contexts overall (Goodman et al., 2020), and there are numerous existing studies on women’s lived experiences of the disease (e.g., Nistor et al., 2023). However, although CD has been shown to predominate in males in mid-late childhood and early adolescence (Targownik et al., 2023) and despite numerous recommendations otherwise (e.g., Sykes et al., 2015), there remains a paucity of research on men’s particular experiences of the disease (Prodgers & Gough, 2021), a trend which has been observed in chronic illness research more widely (e.g., Quaglia, 2020). This is all the more surprising given that traditionally hegemonic masculine ideals have been shown to have a negative impact on men’s mental and physical health (Galdas et al., 2005), leading to a reduction in help-seeking behaviors and a resistance to engaging with the emotional and psychological impact of illness (Gough & Robertson, 2017).

Men with IBD have been shown to have poorer coping skills than women, less medication adherence and are less likely to seek healthcare or engage in self-care (e.g., Targownik et al., 2023). As is widely acknowledged, however, men and masculinities are complex and multifaceted, with other forms of social identity influencing and shaping a diverse range of both harmful and beneficial practices in relation to health (Goodwin et al., 2020; Gough, 2013). Online environments have been shown to be spaces where men may feel more able to talk openly about emotional experiences and personal issues relating to illness (e.g., Hanna & Gough, 2016), and blogging has been shown to provide men with IBD a space for emotional release (Prodgers & Gough, 2021).

The rapid burgeoning of social media platforms and the ease of access to them (Page, 2012) has coincided with a growing research interest in the illness narratives told online (e.g., Stage, 2017). In addition to extended, past-oriented narratives typically found on blogs, what Bamberg and Georgakopoulou (2008) refer to as “big” stories, social media is also able to capture “small” stories: briefer, more fragmented accounts as typically seen on social networking sites (SNSs) such as X (formerly Twitter), Instagram, and Facebook (Melander, 2019). As such, social media offers users the capacity to post illness stories multimodally and episodically (Georgakopoulou, 2018; Page, 2012), making it well placed for capturing the more fluctuating, everyday aspects of chronic illness which may sometimes be difficult to express linguistically (e.g., Prodgers & Gough, 2021). In addition, social media accounts present longitudinal opportunities to trace the unfolding of reported experiences and events in near-synchrony with real time (Page, 2012). This study therefore combines both blog and SNS data to allow for a rich and multi-layered insight into men’s online accounts of CD, offering an opportunity to longitudinally trace how their stories unfold. To do so, we use a dialogical approach (Sullivan, 2012) which centers on subjectivity and experience to enable understanding of how individuals use discourse to negotiate their selves in relation to others. Specifically, we focus on the key dialogical concepts of *forms of authorship, genre* (specifically, the epic and the novel), and *chronotope*, which we argue are particularly useful in highlighting the subtle shifts in self-other dialogues that occur over time in relation to fluctuating disease.

The dialogical emphasis on emotional and embodied truths as opposed to more abstract, theoretical truths (Sullivan, 2012) has led to its increasing use in health-related research contexts (Gomersall & Madill, 2015; Madill & Sullivan, 2010; Thompson et al., 2019a). Originally a form of literary criticism, this approach orients theoretically to Bakhtin’s (1984) dialogism, a relational epistemology in which meaning is constantly exchanged and developed via interaction between different voices. It has since been developed as a qualitative method for use in psychology by Sullivan (2012). Given that individuals typically post on social media in anticipation of a generalized audience (Sullivan, 2012), the dialogical emphasis on the relationship between self and other makes it well placed to explore how *forms of authorship*—the way in which one posts about oneself online—may change across temporal and spatial contexts.^1^ Whilst previous work in health has used a dialogical approach to consider changes in narrative self during a period of illness (e.g., Monforte et al., 2018), we are not aware of any to date that has done so with longitudinal data or with a focus specifically on forms of authorship.

*Genre* (Bakhtin, 1981) refers to the way in which a text is structured around particular stylistic conventions, such as certain patterns of time and space, character types, and so on. This in turn attaches it to particular ideologies and makes certain truths and subjectivities possible. Given that genre can enable exploration of the shaping of self and affective experience via discourse, it has previously been used to provide insight into health-related topics, such as student-doctor experiences in medical training (Madill & Sullivan, 2010). From a Bakhtinian perspective, there are two “grand” genres within which other genres broadly fall—the epic and the novel (Madill & Sullivan, 2010)—which we found to be particularly illuminating in our analysis. Whereas the novel is an anti-authority discourse with a “diversity of truths” and a mixture of voices, the epic genre is monological and authoritative (Sullivan, 2012, p. 60).

Finally, the dialogical concept of *chronotope* (Bakhtin, 1981), which translates literally from the Greek as “timespace,” refers to the intrinsic intertwining of time and space into distinct constellations via genre. It has been shown to be a particularly useful concept for exploring how the relationship between the body, time, and space can shift in relation to illness experience and has previously been used by both Gomersall and Madill (2015) and Thompson et al. (2019b) to examine experiences of long-term health conditions (type 2 diabetes and multiple sclerosis, respectively).

This focus on time and space and the related embodied, lived, and emotionally invested truths therefore make a dialogical approach particularly suitable for addressing the research question: “How do men who post publicly on social media author themselves and their experiences of Crohn’s disease?”

## Method

### Ethics

Ethical approval for this study was granted by the School of Psychology at the University of Leeds. Processes were informed by the British Psychological Society Ethics Guidelines for Internet-Mediated Research (2021). Informed consent was obtained electronically by the first author (LP) and included approval to use images and verbatim quotes from relevant social media posts with clarity that, due to the nature of the material, participants would be personally identifiable in reports of the research. The right to withdraw was detailed and discussed with participants throughout the study both via email and video call. Prior to finalization, participants were given the opportunity to review the analysis of their case and (a) withdraw totally from the study at this point or (b) agree a level of de-identification that they were comfortable with. All three participants agreed to commence without changes.

### The Researchers

The first author (LP) is female, and it was made known to participants in initial contact emails and participant information sheets that she, too, has a diagnosis of CD. The second author (male) (BG) and third author (female) (AM) supervised this research. The latter has a diagnosis of ulcerative colitis which, alongside CD, is a prevalent form of IBD.

Whilst two of the authors having lived experiences of IBD may have had the advantage of shared knowledge and understanding, it brought with it the risk of preconceptions and taken-for-granted assumptions about the disease and participants’ experiences and understandings of it (Mannay, 2010). As Frances (2023) points out, the self is not unitary but dialogical, multiple, and fluctuating. As such, we avoided creating a “shopping list” of positionality statements (Folkes, 2023) and instead continually “dialogued with” the research and our respective roles within it throughout the process. Together and in isolation, this allowed us to subject our ideas, analyses, and assumptions to critical scrutiny and consider how we interacted with and gave shape to this work.

### Participants and Recruitment

Participants were sourced via a systematic Google search deploying key terms including “men,” “blog,” “Crohn’s disease,” and “IBD.” Eastham’s (2011) definition of a public blog was used to identify potential participants such that all social media accounts were open access, open to reader comments or interaction with readers, included identity-revealing photographs, and featured participants’ real names. The selection criteria were that potential participants identified as male; were at least 20 years old at time of recruitment (hence, at least 18 years old when posting material included in the research); had a diagnosis of CD; were fluent in English; and wrote a blog and posted on other SNS, which contained regular posts (at least one per month on at least one platform); were available in the public domain without registration; were not primarily promotional in nature; were written in English; and focused on CD. Three potential participants were selected based on the criteria and contacted, and all consented to take part having read the information sheet and discussed the study with the first author (LP). All are of white ethnicity.

#### Troy

At the time of recruitment, Troy was 28 years old and lived in Canada. He has a keen interest in health, nutrition, and exercise and offers coaching services via his CD fitness blog. He is also a Men’s Physique competitor and a geologist and self-defines as a CD advocate. Following recurrent bacterial infections and episodes of severe pain, diarrhea, and weight loss, Troy was diagnosed with CD in 2009 at the age of 17. He started his blog in 2015, having found little support available when he was first diagnosed, and posts most regularly on his Instagram page, which he started in 2014. In addition, Troy maintains both X and Facebook pages.

#### Nigel

Nigel was 65 years old at the time of recruitment. He lives with his wife of 41 years in the United Kingdom. In 1977, at the age of 21, he started suffering from urgent diarrhea, weight loss, and fatigue and was initially diagnosed with irritable bowel syndrome. Following worsening symptoms, he was admitted to hospital and finally diagnosed with CD in July 1978 at the age of 22. Prior to his retirement in 2017 at the age of 60, Nigel worked in construction as a large project planner. He started his blog in 2010 to keep friends and family updated whilst he was undergoing surgery. He joined X in 2011 where he posts regularly and has had an Instagram account since 2016.

#### Vern

At the time of recruitment, Vern was 52 years old. He lives in Canada with his wife of over 17 years, their two sons, and their dog. He was diagnosed with CD in 1988 at the age of 20 following the sudden onset of symptoms just 6 months earlier. As a young man, and prior to his diagnosis with CD, Vern was a keen athlete, competing in figure skating and active on the soccer field. He was employed by the same company for over 15 years before becoming self-employed as a medical transcriber. Vern’s CD blog has been active since 2009 and he also maintains Facebook, X, and Instagram pages.

### Data Collection

Social media data were collected for each participant constituting posts from their public blogs and active SNS during the 2 years immediately prior to recruitment to the study: specifically from April 2017 to April 2019. It was decided to exclude comments from other social media users, as well as participants’ subsequent responses to them. Inclusion of this data would have significantly increased the overall volume of the dataset. More importantly, however, in a Bakhtinian dialogical analysis (Sullivan, 2012), the role of the *anticipated* other—the audience the author anticipates or imagines dialoguing with—is as, if not more, important for an understanding of subjectivity as that of the *actual* other—the other interlocutor in a dialogue. It was felt that the amount of further insight gained from inclusion of this material would therefore not be warranted pragmatically.

SNS posts were screenshot, and blog data, including images, copied and pasted into three participant-specific MS Word and PowerPoint documents. For ecological validity, original formatting was retained as far as possible. The data was then screened, and content without clear relevance to CD was deleted. After screening, there were 246 pages of data for Troy (11,317 blog words; 133 SNS posts; 128 images), 172 pages for Nigel (29,473 blog words; 400 SNS posts; 114 images), and 421 pages for Vern (21,853 blog words; 512 SNS posts; 364 images).

### Analytical Procedures

A case-by-case and idiographic approach was adopted to retain the integrity of each participant’s material and, from this basis, to allow analytical comparisons to be made between cases (de Visser & Smith, 2006). In the first exploratory stage, each participant’s dataset was read through several times and observations noted regarding phenomena of particular interest to addressing the research question. This enabled the data for each case to be organized into descriptive categories on an Excel spreadsheet cataloguing each individual blog and SNS post. Extensive hand-written notes were then made in relation to each post, and connections between posts were identified to aid the development of a cohesive case study for each participant. In the second stage, a top-down approach was adopted in which the key Bakhtinian concepts outlined above, that is, genre, chronotope, and forms of authorship, were used to orient the analysis of each case, following Sullivan (2012). Specifically, during analysis, the genres of epic and novel were found most helpful in characterizing the material, their use, and juxtaposition distinguishing the cases analytically. Writing occurred continuously throughout the process, and analyses were conducted iteratively by cycling drafts between the first and co-authors to instigate discussion to help clarify and refine the analysis.

## Analysis

Three case studies are presented focusing on the dialogical concepts of genre (epic and novel), chronotope, and forms of authorship. As will be seen, all three participants authored themselves and their CD in relation to chronotopes associated with uncertainty, disruption, and fragmentation but each responded to this in different ways. Images from the online posts are included where relevant and integrated into the analysis. The symbol […] is used to denote where, for efficiency of exposition, some text has been omitted mid quote.

### Case 1: Troy

Troy’s social media privileges an epic authorship in which he is the hero working toward conquering his disease:

Knowing how far I’ve come, what it’s like to hit rock bottom and battling my way back to a better version of myself than I was before Crohn’s is what keeps me moving forward. Consistently pushing myself beyond what I thought I was capable of. (Blog excerpt)

**Figure 1.**
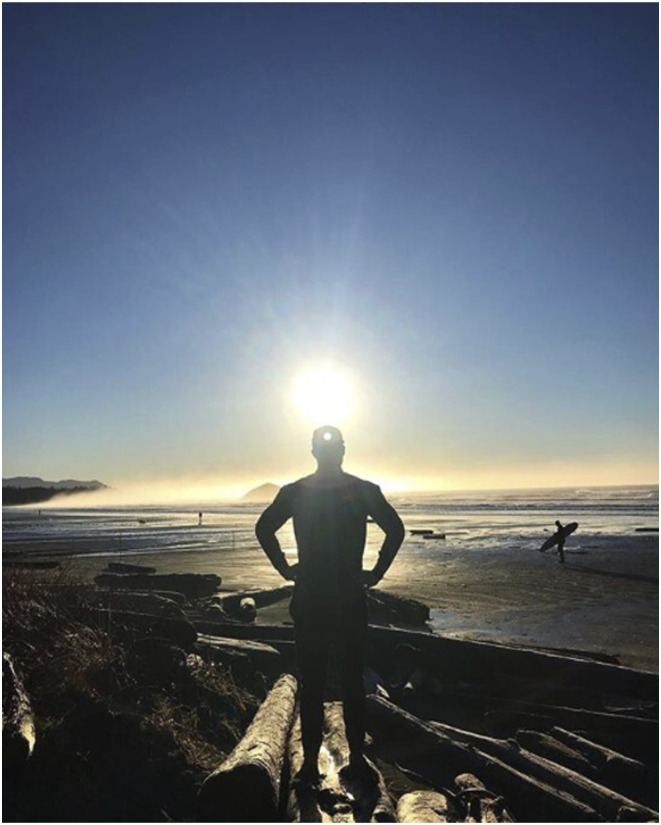
Image of Troy from Instagram in an epic outdoor landscape.

Although Troy has pushed himself “beyond what I thought was capable of,” the “better version of myself” that he acquires appears, to use Bakhtin’s (1981, p. 36) phrase, “ready-made”: that is, pre-determined. In these posts, the chronotope combines “fixed, stage-like and formulaic” time with a certain future (Sullivan, 2012, p. 46) with grand outdoor landscapes and spaces of bodily improvement, such as the gym. These spaces retain distance from the audience to create a sense of heroic spectacle (Figure 1).

**Figure 2.**
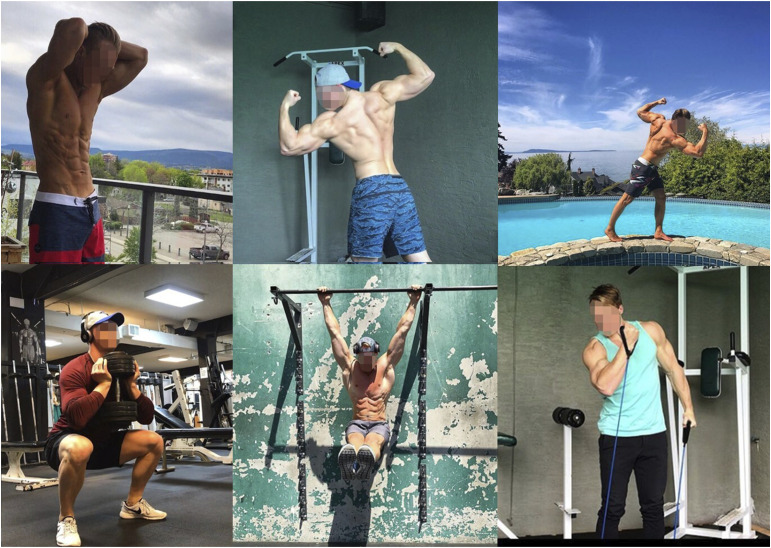
Images of the “epic hero” from Troy’s Instagram.

As seen in Figure 2, Troy frequently includes images of himself within these spaces using medium close shots (showing himself from the waist up) and far personal distance (in which, if in person, he would be just out of reach) (Kress & van Leeuwen, 2006). In so doing, he invites the audience into an intimate view of his partially clothed body, whilst holding them at enough of a distance that he is—both literally and figuratively—untouchable. At the same time, he maintains a distance from himself as the experiencing subject and his day-to-day lived realities: what is witnessed is perfected and polished, not gritty reality. For instance, in a video posted following his surgery, he notes, “if you look closely, the scar below my belly button constantly reminds me of the adversity I’ve faced and the struggles I’ve had to overcome” (blog excerpt). The tiny scar on his abdomen is liable to be overlooked without his explicit acknowledgment. Juxtaposed against its muscular backdrop, Troy actualizes an excess of physical strength whilst minimizing the impact of his Crohn’s. As such, he remains in control of his destiny: there is no openness to be shaped or transformed in alternative ways by his disease or by his audience. He is “Conquering Crohn’s 1 rep at a time” and is “Fuelled by adversity” given “Nothing feels better than healthy feels” (Instagram excerpts). Therefore, his heroic battle simply continues on “towards complete optimization of my physical and mental health” (blog excerpt).

**Figure 3.**
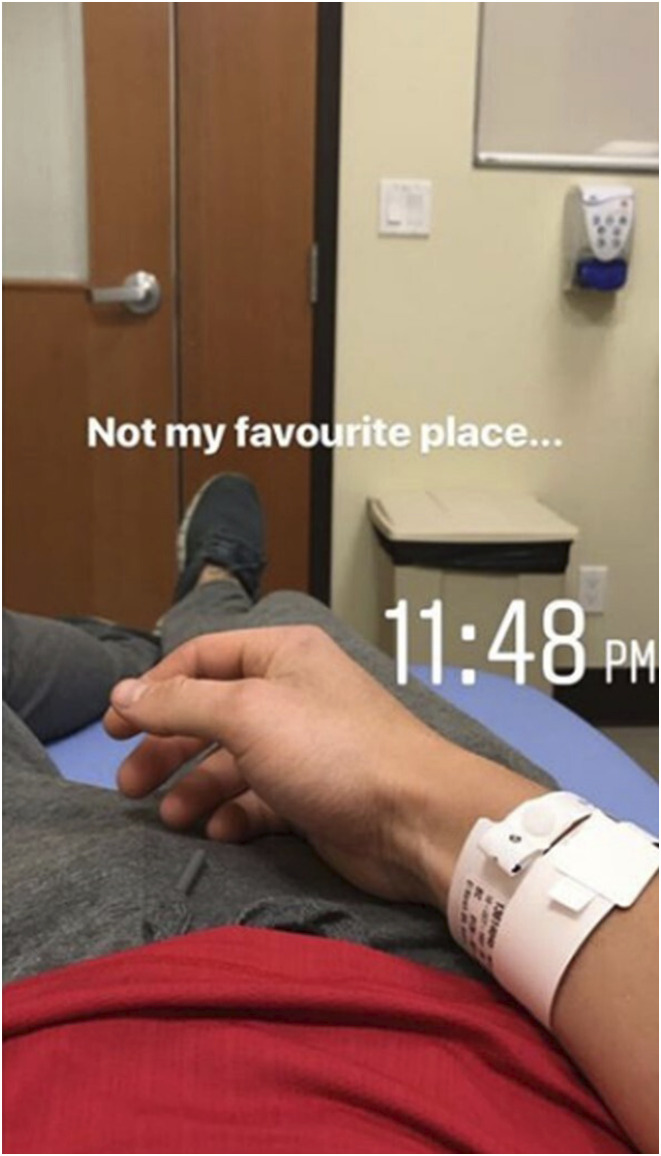
Inferred selfie from Troy’s Instagram.

There are, however, fleeting moments when Troy employs a more novelized genre on his social media. Here, the chronotope shifts to more intimate, personal spaces, such as the hospital, as he transitions from photographs taken *of* him to self-taken images or, to use Zhao and Zappavigna’s (2018) term, “inferred selfies.” In these, the subject’s face cannot be seen, but their presence is implied in the appearance of their body parts (Zhao & Zappavigna, 2018). Such images allow the viewer to picture a scene from the perspective of the photographer, inviting them to share in the experience (e.g., Figure 3). Time also becomes more uncertain in these moments, moving away from an idealized past and certain future to a focus on the present moment, bringing the audience closer to his lived realities: “Despite my best efforts to control my disease through medication, supplementation, nutrition and overall wellbeing, I do not have complete control over my disease” (Instagram excerpt).

Whilst these fleeting novelized posts appear to make Troy more relatable to his chronic illness audience and thus offer him connection with an understanding and supportive online community during difficult periods—“The amount of support the last few days has been overwhelming […] A huge thanks to the IBD community for coming together and supporting me. It truly means a lot” (Instagram excerpt)—his preferred epic form of authorship appears to produce a sense of certainty and control over his future in the face of stressful destabilization caused by his disease. By celebrating his strength in confronting the challenges of his CD to date, his epic narratives act as reminders of his ability to overcome future health-related challenges. Furthermore, by reframing more vulnerable, emotional, and uncertain aspects of illness in a more positive and even awe-inspiring light, his social media posts seem to have both affirmative and protective functions.

### Case 2: Nigel

**Figure 4.**
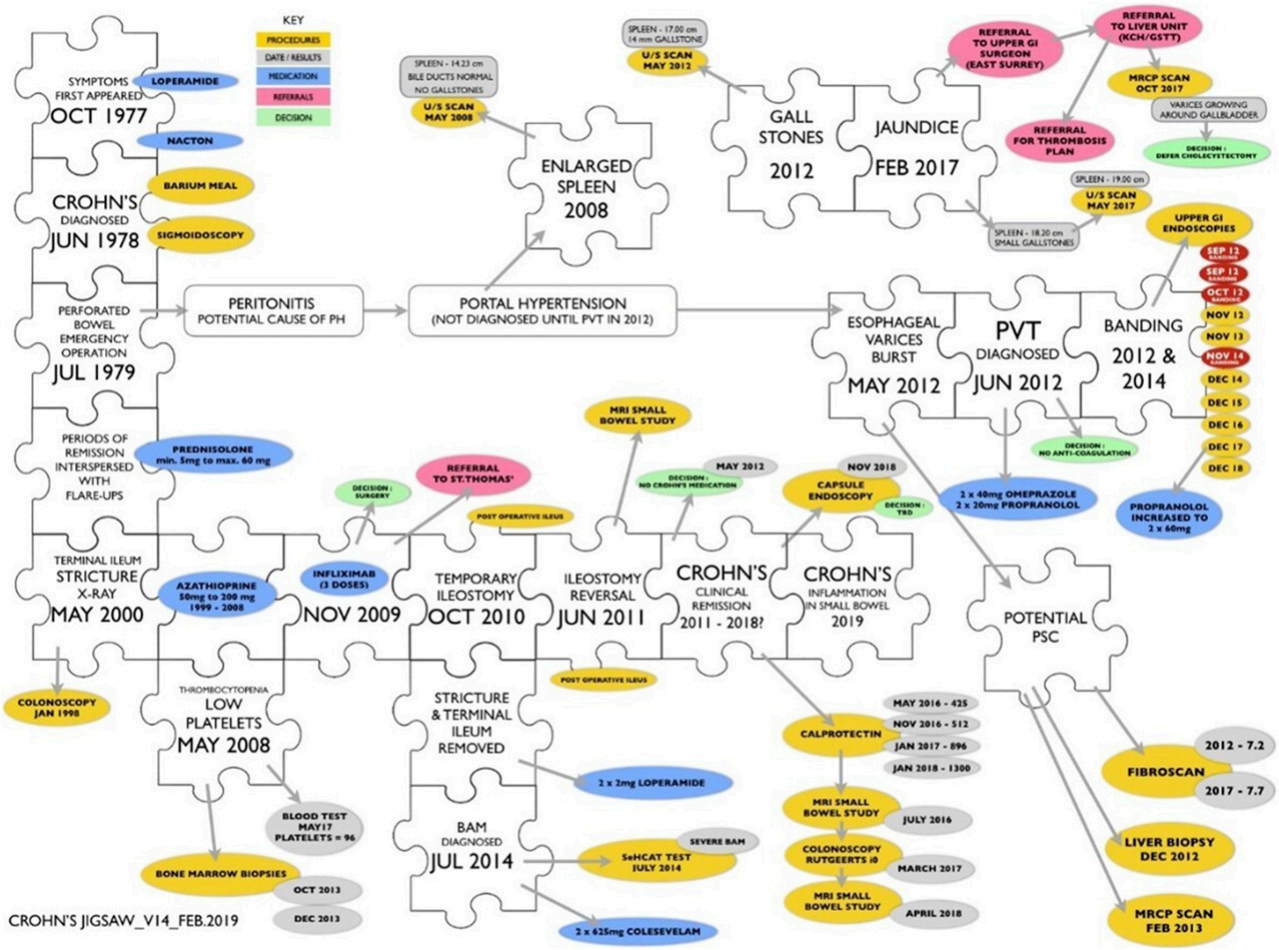
Nigel’s jigsaw diagram of his health puzzle which appears across his social media.

In Nigel’s social media, the dominant genre used is again the epic, this time an epic mission in which Nigel as the hero-protagonist is challenged to restore a state of equilibrium following its disruption by an unruly outside force (Posman, 2006). For Nigel, cyclical time, an extended period of relatively settled disease consisting of “remission interspersed with flare-ups” (blog excerpt), is disrupted via the exposure of “a complicated picture” (blog excerpt) of multiple hidden complications of disease and new diagnoses of which he was previously unaware. He therefore writes as the archetypal detective, using his social media as a space in which to make his body more knowable and restore it to order: “My challenge today is to link (in no particular order): an unresolved medical test; distinguishing between the effects of long-term medication and the ageing process; another meeting with the surgeon and overcoming the stomach-churning effect of burnt bananas” (blog excerpt). To do so, time is typically conceptualized in epic fashion as teleological and goal-oriented and the body is spatially transformed into pragmatic puzzles, charts, and data. These corporeal conceptualizations bring to light the gaps which still needed to be filled to complete his health puzzle. This means creating distance from himself as the experiencing subject and, instead, taking an objective stance firmly “outside” the body. This is typical of epic authorial discourse in which the hero (in this case, Nigel as patient) is tightly controlled by the author’s (Nigel as detective) voice (Sullivan, 2012). This is further illustrated in Nigel’s jigsaw diagram constructed from over 40 years of medical records (Figure 4):

I drew it, initially, to try and understand the relationships/causes between the various conditions I have ended up with. It then dawned on me that it would [be] a good way of showing a new doctor or surgeon the complexity of my case on just a single page. (Blog excerpt)

Using the diagram, Nigel is able to view his CD from the outside-in, “occupying an intently maintained position *outside* the hero” (Bakhtin, 1990, p. 14). Such distancing enables an emotional disengagement with the felt reality of his increasingly complex ill health which “can only get worse as the ageing process kicks in” (blog excerpt). Much like Troy, this epic genre appears to offer Nigel certainty in the face of disruption. By simplifying and reducing the space of his medical history whilst creating complex and networked temporal clusters of medical events in the diagram, he is able to reassuringly fill in the “gaps” of his own and medics’ knowledge and understanding of his case. Implicit within the jigsaw metaphor is also the idea of completion: its fate, much like the epic hero’s, is pre-destined. In the face of unreliable outer lived experiences (his previous lack of awareness of growing health issues), Nigel can thus distance from himself as the difficult and unreadable patient to assess and analyze his inner body from one step removed: “I [tried to] spot any correlation between the drug dose and the platelet count […] The only way to investigate further was to have a bone marrow biopsy” (blog excerpt). Making himself more “knowable” perhaps offers a sense of coherence and temporary reassurance in the face of ambiguity.

Running counter to the archetypal detective is a more novelized version of Nigel’s detective. Across his social media, tensions are revealed as the more he learns about his health, the more gaps become exposed and the more unknowable the body and/or the riskier these discoveries become: contrary to the jigsaw’s implicit function as a puzzle to be completed, it in fact *resists* completion. For example, after months of tests and investigations to explore his ever-rising inflammation levels, it is revealed that Nigel’s CD is active again: a possibility he had previously refuted due to a lack of external symptoms. Nigel posts:

I thought more about why I was surprised [about having active Crohn’s] and concluded that I really shouldn’t be. Colonoscopies always showed no inflammation; upper GI [gastrointestinal] endoscopies showed the same. It was only the elevated calprotectin level that suggested anything was wrong. If that level wasn’t a false positive then the problem had to be somewhere between the duodenum and the terminal ileum. The last small bowel MRI scan had mentioned the possibility of inflammation. (Blog excerpt)

When taking into account the empirical evidence as any good objective detective should, only one conclusion could be arrived at: “the problem had to be” active CD. Yet, rather than following the clues to their logical conclusion, he had willfully avoided the latter until “it was in black and white” (blog excerpt). The reality of active disease thus appears to be an answer he wishes to evade, perhaps unsurprisingly given his previous life-changing complications from certain medications, the complexity of his prior surgery, and the risk inherent in such treatment for older CD patients: “If we [‘elderly’ IBD patients] need emergency surgery we have a 1 in 4 chance of not surviving it!” (X excerpt).

Nigel’s posts thus demonstrate a contrast between his curiosity as the objective detective (wanting to know) and the retrospective wish to un-know as the experiencing subject (not wanting to know). Writing as the detective, he can distance from the body and reduce it into a series of mere “clues” to rationalize, observe, and fill in the gaps of his health. As the experiencing subject, however, he is confronted by the inescapable nature of his subjective, embodied reality and the limits of medical knowledge to resolve his increasingly complex health issues.

### Case 3: Vern

Whereas Troy and Nigel privilege epic narratives which emphasize the need for certainty contra the way in which this is challenged by their CD, Vern privileges more novelized narratives allowing for unexpected changes over the 30 years since the onset of his illness. His social media are organized around two main overarching narrative time structures. The first is of rupture, as experienced in “threshold moments” constituting instances of “crisis and break in a life” (Bakhtin, 1981, p. 248). These lead to significant psychological and physical transformations, fundamentally shifting his embodied sense of being in the world. The second is of cycles, in which everyday life is repeatedly and unexpectedly disrupted by relapses of illness symptoms.

In threshold moments, which occurred at the onset of his disease, Vern’s life changed irrevocably from the stability of cyclical rhythms of time and familiar settings of the everyday due to traumatic transformations in which time becomes abruptly destabilized and familiar spaces, including those of the body, become strange and alien:

One morning I got up, showered, shaved had breakfast and laid on the couch to watch the news before work as usual […] As I watched the news I started to feel something in my throat […] kept swallowing and swallowing to try and get it to move but to no avail […] I could feel it move down my throat, into my chest and into my stomach, then the feeling I had to go to the washroom and the sudden urge to have a bowel movement. And that’s where the pain started. Just like that. No inclination of a problem. Nothing leading up to it. No signs. Nothing. (Blog excerpt)

**Figure 5.**
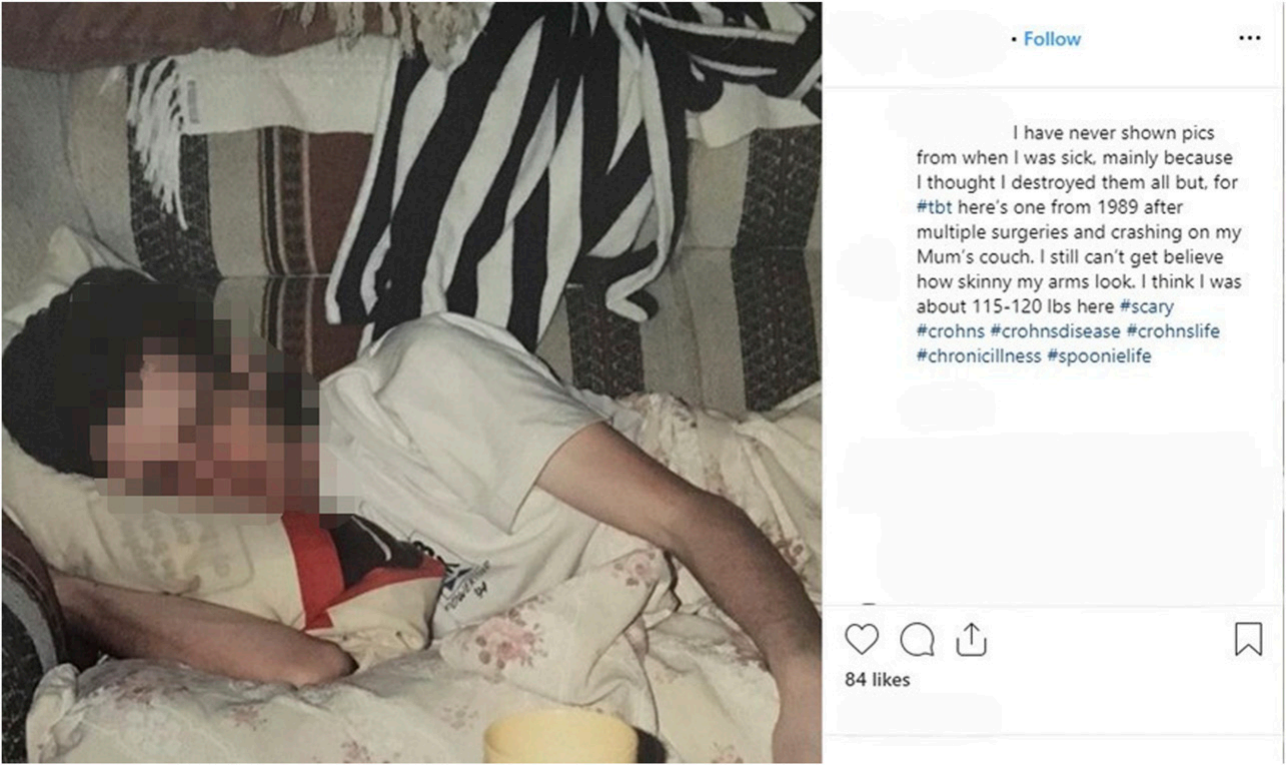
Image of Vern when recovering from surgery in 1989, taken from Instagram.

As his CD symptoms appear abruptly and dramatically as though from nowhere—“just like that”—Vern shifts swiftly from the routinized and familiar to “instantaneous” time (Bakhtin, 1981, p. 248). His sense of surprise and shock are palpable in the absence of any prior symptoms and, as has been reported elsewhere in patient descriptions of pain (e.g., Kirkham et al., 2015), there is a horrific sense of being taken over by an alien entity as he is literally consumed from the inside out: “Crohn’s disease ate my colon” (blog excerpt). This threshold moment brings with it a shift in his experience of the domestic space, once a site of familiarity and stability but now one of involuntary imprisonment (Figure 5).

Despite depicting a scene of apparent relaxation, the text accompanying Figure 5 includes an undertone of violence which speaks to Vern’s changed emotional state. He notes that “I have never shown pics from when I was sick […] I thought I destroyed them all” (Instagram excerpt). Echoing the horror of his onset of symptoms, Vern labels the photo as “#scary” and indicates that several decades later, he still finds the way he looked hard to comprehend: “I still can’t […] believe how skinny my arms look” (Instagram excerpt). Once the everyday guy, he authors himself as the victim of an unexpected, traumatizing, and life-changing rupture: “that was the last time life would be ‘ordinary’” (blog excerpt).

More recently, Vern indicates that life is still punctuated by ruptures as he experiences repeated “chronotope disruptions”: biographical disturbances in the experience of time and space (Gomersall & Madill, 2015). During these episodes, there are echoes of the threshold moments of his past as “just like that” (blog excerpt) the everyday becomes unexpectedly disrupted:

Monday was the same as any other, nothing out of the ordinary […] Around 2am I got up to go to the bathroom […] I started to feel chilly […] I started to get the shakes and shivers […] after a good few minutes I knew it was the start of rigors […] This past attack lasted about 2 hours and, like always, after I threw up, the shaking started to slow and then eventually … just … stop. (Blog excerpt)

In comparison to the impact of the original threshold moment which continued for months causing physically transformative effects, these chronotope disruptions are more fleeting. After reaching their peak, usually within a matter of hours, Vern’s various bodily metamorphoses wane and “eventually … just … stop.” Whilst usually appearing out of nowhere, then, there is something of the routine to these episodes: “like always” the symptoms start to ease. As such, whilst not anticipated in the actual moment of occurrence, these disruptions *are* now expected in the longer-term cycle of his chronic illness: life is more certain and reliable with its cyclical rhythm of fluctuating phases of flare and recovery.

Therefore, where Vern authors his past self as a victim taken by surprise, in the present he authors himself as the knowing expert. This appears in part thanks to his contemporary access to social media. In contrast to his early days of disease during which “Crohn’s made me a virtual shut in” (blog excerpt), now he has access to a social space even when he is physically isolated due to disease symptoms:

Fast forward 30 years [from my diagnosis] and there are web pages, blog pages, vlogs, etc. out there with a ton of information. Facebook, Twitter, Instagram, etc. help others living with the disease connect talk and share experiences […] I remember being feeling so isolated and alone suffering through the pain and embarrassment of the disease. (Blog excerpt)

**Figure 6.**
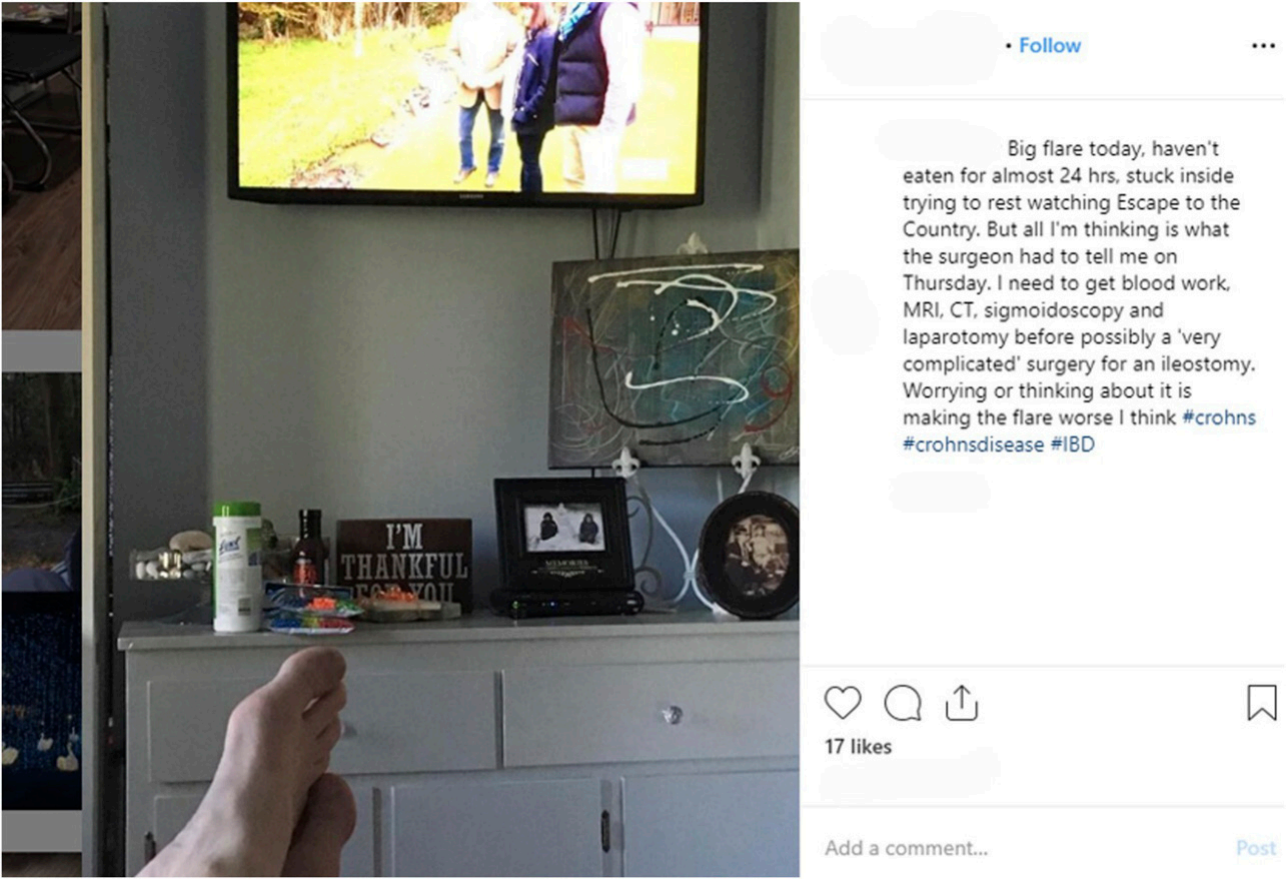
Inferred selfie from Vern’s Instagram.

Whilst Vern may be physically “shut in” during moments of chronotope disruption in the present day, he is no longer socially “shut in” as he is able to benefit from a sense of community, reciprocity, and connection online. This is exemplified in his use of inferred selfies during periods of recovery from flare-ups of disease (Figure 6). Vern finds himself involuntarily “stuck inside” the domestic space and, concomitantly, the internal space of his mind: “all I’m thinking is what the surgeon has to tell me.” However, rather than simply ruminate on his concerns, social media allows him to disclose his thoughts, freeing him from the sense of mental imprisonment he once had when he was “keeping everything in” (blog excerpt).

Vern’s social media posts therefore highlight his changing emotional response toward the uncertainty caused by his disease. Where once it was traumatizing, leading to a total change in his embodied and psychological experience and resulting in his social isolation, now, with a sense of lived expertise, any frustration, anger, and sadness are short-lived. Thus, whilst his CD clearly continues to create disruption, life overall is written as having greater stability and security. This is further bolstered by his use of social media which, when he finds himself suddenly unwell, offers a bridge between the inner domestic space and outer spaces of productivity, reducing his sense of social isolation.

## Discussion

Using a dialogical approach, this study addressed the research question: “How do men who post publicly on social media author themselves and their experiences of Crohn’s disease?”. Whilst all of the participants showed a preference for certain types of authorship, they authored themselves in various ways across their social media. This often differed according to disease activity and/or illness-related events happening in the material world. Timespaces associated with (un)certainty and disruption/order were common, emphasizing the lack of predictability of CD and the resultant impact of this on the participants’ lives and embodied experiences. This lack of certainty has previously been reported to be problematic for many with long-term illnesses (e.g., Brown et al., 2020) and CD in particular (Niv et al., 2017). Overall, in the face of this uncertainty, posting on social media appeared to offer a greater sense of control, be it over their sense of embodiment and self-image (Troy), their knowledge and understanding of their medical histories (Nigel), or in their interactions and connections with others (Vern).

When posting online, men have typically been shown to be more task-oriented and motivated by information gathering (Krasnova et al., 2017) and less likely than women to receive social support (Tifferet, 2020). Whilst this appeared to be the case for both Troy and Nigel, the study’s in-depth analysis indicated that social media had a more subtle support function for them both, particularly at times of active disease or when faced with health ambiguity. Vern’s narratives more explicitly emphasized support functions. These findings add nuance to the existing literature which indicates that online chronic illness communities can be important sites of social and emotional support and solidarity, particularly during periods of ill health (Berard & Smith, 2019).

By distinguishing the subtle variations in participants’ construction of experience over time in relation to, for example, different levels of disease activity and different modes of post, the study also provides understanding of within-person variability in relation to men’s vulnerability and conformity to masculine norms. This has been highlighted by Schwab et al. (2016) as integral to an understanding of masculinities as neither fully conforming nor non-conforming to hegemonic ideals. Vern was open about his emotional vulnerabilities and more accepting of marginalized masculine positions, such as in his self-authorship as the passive CD victim. The nature of his vulnerability changed between the period of initial diagnosis and the present day, however.

Troy’s preference for the epic hero positioned him as rational, autonomous, and action-orientated, stereotypically masculine traits which are often used to frame positive approaches to health promotion (Sloan et al., 2010). However, his more novelized forms of authorship on social media brought to the fore the mixture of emotions he experiences and changing masculine positions he adopts during episodes of more acute illness. Nigel’s archetypal detective and construction of a controlled outer body drew on hegemonic masculine signifiers of emotional and psychological resilience, as well as autonomy (Addis & Mahalik, 2003; Smith, 2013). However, his movement between the objective and subjective detective exposed how maintaining a balance of medical knowledge—neither knowing too much nor too little—was fundamental to his maintenance of emotional balance.

A unique contribution of this study was therefore that it emphasized how the participants made sense of the different and ambiguous ways in which they *experienced* these contradictory discourses and how their investments in them changed according to context. Whilst a theoretical understanding of constructions of masculinity in the participants’ narratives and dialogues could be identified—what Bakhtin (1993) would call the “istina,” an abstract form of truth—the focus was brought instead to the “pravda,” the lived truth. In other words, the embodied and emotionally invested truths that participants attached to such constructions were attended to over and above abstract ideas of masculinity (Sullivan, 2012). The absence of the latter has often been considered a shortcoming in discourse analytic work which focuses on linguistic practices, action-orientation, and power relations over subjectivity and emotional experience (Gough, 2004). However, participants’ words were also not treated as offering straightforward insight into their inner worlds, as in phenomenological approaches (Sullivan, 2012). Rather, we drew attention to the ways in which participants made sense of themselves and their (masculine) identities in relation to their health struggles, but also how the meanings they constructed were often ambiguous and various, giving the sense of a conflicted self.

As Ressler et al. (2012) have pointed out, blogging in near-synchrony with real time can emphasize the evolution of understanding in individuals’ accounts of illness experience as they seek a sense of purpose and meaning from disorder and hopelessness. Social media’s propensity for ongoing storytelling (Bamberg & Georgakopoulou, 2008) meant that, in this study, the fluid and changing ways participants constructed their narratives over time could be identified, and aspects of their illness experience which resisted conventional narration could be captured and understood as part of the broader picture of their overall ongoing experiences. Given many chronic illnesses involved experiences of fragmentation and disruption, such an approach could be fruitfully applied to other conditions to gain a deeper and multi-layered understanding of lived experiences, enabling insights which more “traditional” methods may not capture.

Furthermore, as Monforte et al. (2018) point out, stories are a source of knowledge. Johnson et al. (2020) have recently explored the potential of applying narrative therapy in conjunction with social media use as a narrative therapy technique for active social media users living with chronic illness. With ethical safeguards in place, the careful curation of social media narratives of chronic illnesses as have been produced as part of this study could also have numerous uses. In therapeutic contexts, these could be used as a means to engage individuals with others’ understandings and experiences of the same condition, offering a starting point from which to discuss and potentially reformulate their own. For charities and others wanting to raise awareness and understanding of a certain condition, such curated narratives offer a way to engage the public in an empathic, thought-provoking, and multimodal way. Further research into the possibility and potential challenges of these approaches would therefore be worthwhile.

Whilst the focus on participants’ embodied and emotionally invested truths allowed for consideration of the emotional and experiential dimensions of masculinity, the role power played in participants’ narratives remained underexplored. This was particularly significant given the relative power granted to participants in the intersection with their other identities as white, cis-gendered, and heterosexual. Given the experiences of minoritized ethnic and non–cis-gendered groups with CD are currently underexplored (Alexakis et al., 2015; Targownik et al., 2023; Veracruz & Taft, 2022; Windsor et al., 2023), further research is required to develop better understanding of these particular experiences and the impact of imbalances of power on them. Moreover, the internet also restricts and discriminates (Peterson, 2009). Such research inevitably overlooks those who do not have the means, the desire, or, indeed, the capacity to post on social media. Given so few men in general appeared to write blogs about their experiences of CD and the fact that, of those identified that did, all were from majority ethnic, sexuality, and gender identity groups, it is important to explore this phenomenon further asking, whose stories are typically told online, which stories are seen as acceptable, and which are routinely missed. Finally, and in line with this, whilst for some social media may offer a place of retreat during acute episodes of ill health, it is likely that for others it may offer the opposite. This area requires further research to understand the negative impact of social media use for those with chronic health conditions.

## Conclusion

This study provided unique and valuable insights into three men’s online narratives of CD across a 2-year period. By exploring each participant’s unique engagement in social media in relation to their illness, we have deepened understanding of this currently under-studied area. Combining data from across participants’ blogs and SNSs enabled the development of more nuanced and multifaceted insight into illness stories in action. In so doing, we have distinguished subtle variations in participants’ construction of experience over time in relation to different levels of disease activity and different modes of post. Furthermore, by focusing on the embodied and emotionally invested truths of participants’ narratives and constructions of self and other, we have gained insight into the experiential and emotional dimensions of their changing identities. This has important implications for healthcare practice. For example, heightened awareness of the subtle fluctuations in how men author their illness experiences may be a good indicator of their changing support needs. Furthermore, whilst on the surface one may author themselves and their disease in distanced and seemingly unemotional ways, it is important to recognize that this may in fact be an indication of greater distress: the need to gain control over otherwise overwhelming experience.

## Note

1. It is important to note that the nature and role of the author is the subject of considerable debate in the field of literary studies. Whereas much modern literary theory has treated the author as largely, if not entirely, irrelevant to a text’s meaning (Stougaard-Nielsen, 2019), for Bakhtin (1990), the relationship between author and hero is central to an understanding of the relationship between self and other. In autobiographical writing, one is both the “writing” subject (the author) *and* the “written” subject (the hero) who is “narrated through the eyes of an internalised other” (Erdinast-Vulcan, 2018, p. 417).
